# Bioactive Constituents of Ginseng in Cancer Therapy: Molecular Mechanisms, Translational Potential, and Advanced Delivery Strategies

**DOI:** 10.7150/ijbs.136955

**Published:** 2026-09-03

**Authors:** Jia-hui Li, Yan-fang Xian, De-wen Liu, Hong-yuan Li, Wei Li

**Affiliations:** 1 College of Chinese Medicinal Materials, Jilin Provincial International Joint Research Center for the Development and Utilization of Authentic Medicinal Materials, Jilin Agricultural University, Changchun 130118, China.; 2 School of Chinese Medicine, Faculty of Medicine, The Chinese University of Hong Kong, Shatin, N.T., Hong Kong SAR, China.; 3 Institute of Chinese Materia Medica, China Academy of Chinese Medical Sciences, Beijing 100700, China.

**Keywords:** ginseng, ginsenosides, cancer therapy, anticancer mechanisms, drug delivery, translational potential

## Abstract

Cancer remains a major global health challenge, highlighting the urgent need to develop safer and more effective therapeutic drugs. Ginseng (*Panax ginseng* C.A. Mey.), as a medicinal plant widely used in traditional Chinese medicine, has attracted much attention for its extensive anticancer potential and a variety of biologically active ingredients (especially ginseng saponins and polysaccharides). This article reviews the latest research progress on the pharmacological activity and molecular mechanism of ginseng extract in cancer treatment, focusing on its inhibition of tumor growth, inducing cell apoptosis, inhibiting epithelial-mesenchymal transformation and metastasis, reshaping the tumor microenvironment, enhancing antitumor immunity, regulating metabolic reprogramming, and regulating epigenetics. In addition, this article also explores their potential to overcome multi-drug resistance and enhance the efficacy of traditional chemotherapy drugs. In recent years, a variety of drug delivery systems, including polymer nanoparticles, lipids, and micelles, have been developed to improve the bioavailability, stability, and tumor targeting of these compounds. This article also focuses on the current progress and challenges in clinical transformation, especially in the development and application of ginseng saponin preparations. In a word, this article provides a comprehensive review of ginseng-derived drugs from the perspective of pharmacology and translational medicine, and looks forward to the future direction of their reasonable development in cancer treatment.

## 1. Introduction

Cancer is one of the leading causes of morbidity and mortality worldwide, and is still a huge public health and socio-economic burden. It is caused by the accumulation of genetic and epigenetic changes in somatic cells, leading to uncontrolled proliferation, local invasion, and metastatic diffusion^1^. In 2022, an estimated 20 million new cancer cases and 9.7 million cancer-related deaths were reported globally, and the annual number of new cases is projected to reach 35 million by 2050^2^. Common types of cancer include breast cancer, lung cancer, colorectal cancer (CRC), and prostate cancer (PCa), which still account for a large proportion of cancer incidence and mortality. In view of the increasing global burden of cancer, the development of safe and effective therapeutic drugs remains a top priority in the field of oncology^3^.

Traditional treatment strategies, including chemotherapy and radiotherapy, have improved the clinical efficacy of many cancers, but their application is often limited by systemic toxicity, treatment-related complications, and drug resistance^4^-^7^. In recent years, immunotherapy and targeted therapy have expanded the field of treatment; however, these methods still face many challenges, such as high cost, limited patient response, adverse reactions, and acquired drug resistance^8^. These limitations have prompted people to continue to pay attention to new anticancer drugs and complementary treatment strategies that are safer, more applicable, and more flexible.

Natural products have long served as an important source of anticancer agents, and traditional Chinese medicine (TCM) has received increasing attention in this context^9^. Owing to its multitarget characteristics and systemic mode of action, TCM has been explored not only as a source of bioactive compounds, but also as an adjunctive approach for alleviating treatment-related toxicity, improving quality of life, and supporting comprehensive cancer management^10^, ^11^ (**Fig. 1**). More and more evidence shows that natural products derived from TCM can interfere with many aspects of tumor progression, including proliferation, apoptosis, angiogenesis, metastasis and tumor microenvironment regulation^12^-^14^. In particular, *Scutellaria baicalensis* Georgi (Huangqin), *Sophora flavescens* Ait. (Kushen), *Salvia miltiorrhiza* Bunge (Danshen), *Ganoderma lucidum* (Curtis) P. Karst*.* (Lingzhi), and *Curcuma longa* L. (Jianghuang) have exhibited notable anticancer potential in various *in vitro* and i*n vivo* experimental models^15^-^19^. These medicinal plants provide important resources and a theoretical basis for the development of novel antitumor agents.

Among these candidates, ginseng (*Panax ginseng* C.A. Mey.) has attracted much attention because of its broad-spectrum anticancer properties. Traditional pharmacology shows that ginseng has the effect of toning qi and blood, stabilizing the pulse, nourishing the spleen and nourishing the lungs, and nourishing the blood^20^, ^21^. As a highly valued herb in TCM, ginseng and its active components, such as ginsenosides, polysaccharides, and volatile oils, have been reported to regulate multiple biological processes involved in cancer development, including cell proliferation, metabolism, migration, invasion, and angiogenesis^22^, ^23^. In particular, ginsenosides and ginseng polysaccharides (GPS) have shown great potential in regulating tumor immunity, enhancing immune surveillance, and reducing toxic side effects associated with traditional anticancer therapies (such as bone marrow suppression and organ damage)^24^, ^25^. This dual potential of both antitumor activity and auxiliary therapeutic effect makes ginseng a promising candidate drug in comprehensive cancer treatment.

Recently, several reviews have discussed specific applications of ginseng-derived compounds in oncology, including their combination with chemotherapy^26^, potential role in cancer cachexia^27^, activity against cancer stem cells^28^, and application in ginseng-based nanotherapeutic systems^29^. These studies have substantially expanded our understanding of individual therapeutic contexts. Even so, a comprehensive synthesis that integrates chemical diversity with mechanistic evidence, resistance modulation, formulation strategies, and clinical translation has yet to emerge. Here, we provide such an integrated overview. The available evidence is organized around key biological processes that govern tumor initiation, progression, and therapeutic response, allowing a more mechanism-oriented interpretation of the field. We further compare the level of experimental support across different classes of ginseng constituents and examine how pharmacokinetic characteristics influence delivery design and clinical applicability. This review summarizes current knowledge of the pharmacological activities and molecular mechanisms underlying the anticancer effects of ginseng and its major bioactive constituents. It also discusses recent advances in clinical investigation together with innovative drug delivery approaches aimed at improving efficacy and bioavailability. Finally, we consider the major barriers to clinical translation and outline future directions that may facilitate the development of ginseng-derived compounds as effective anticancer therapeutics.

## 2. Chemical components of ginseng

Ginseng is a well-known medicinal herb in TCM with a long history of clinical use. It contains a wide range of chemical constituents, including ginsenosides, GPS, volatile oils, proteins, amino acids, and peptides^30^. In addition to the traditionally used roots and rhizomes, ginseng stems and leaves are also rich in ginsenosides and other bioactive or nutritional constituents, highlighting their potential as underutilized resources for the development of ginseng-derived products^31^. Among these, ginsenosides and GPS are generally regarded as the major bioactive components associated with the pharmacological activities of ginseng, including its anticancer effects^32^.

Ginsenosides are the principal active constituents of ginseng and exhibit diverse pharmacological properties, including anti-inflammatory, antioxidant, antitumor, and antifatigue activities^33^. To date, more than 140 ginsenoside monomers have been isolated and identified from ginseng. Based on differences in their aglycone structures, ginsenosides can be classified into protopanaxadiol (PPD), protopanaxatriol (PPT), and oleanolic acid (OA) types. From a pharmacological perspective, these compounds are further distinguished as prototype or rare ginsenosides according to their abundance and metabolic origin^34^. Rare ginsenosides have attracted particular interest in oncology. Among them, Rg3 and Rh2 are the most extensively studied, owing to their potent anticancer activities and increasingly well-defined mechanisms of action. Rg3 has entered adjunctive clinical use in China as a component of several anticancer preparations^35^. **Fig. 2** and **Table 1** provide an overview of the classification of ginsenosides, the major scaffold structures, and the interconversion between the major saponins.

GPS are another important class of bioactive constituents. According to monosaccharide composition, GPS can be broadly divided into neutral polysaccharides and acidic pectic polysaccharides. Neutral fractions mainly include glucans and arabinogalactans, whereas acidic fractions are typically rich in galacturonic acid^36^. In addition to their antioxidant properties, GPS have been reported to exert immunomodulatory and antitumor effects. These activities are thought to be mediated, at least in part, through the activation of immune cells and the enhancement of immune surveillance, thereby contributing to the anticancer potential of ginseng^37^.

## 3. Biological mechanisms of ginseng-derived constituents in cancer therapy

Cancer progression is increasingly recognized as a dynamic systems-level process rather than the consequence of isolated molecular abnormalities. Continuous proliferation, resistance to programmed cell death, metastatic plasticity, immune escape, metabolic adaptation, epigenetic remodeling, and interaction with the host microenvironment jointly determine the evolution and therapeutic response of tumors^38^. These interrelated biological processes provide a useful framework for understanding the pharmacological effects of ginseng extract, whose reported activity goes far beyond direct cytotoxicity.

Among the many components that have been identified in ginseng plants, research on the mechanism mainly focuses on ginseng saponins. Rare saponins, especially Rg3, Rh2, Rg5, Rk1 and Rk3, as well as intestinal microbial metabolite compounds K (CK), constitute the field with the richest experimental evidence. In addition, people are also paying more and more attention to GPS, glycosides, chemically modified derivatives, and standardized plant preparations, which occupy a unique position in the field of pharmacology. Importantly, the functions of these compounds should not be considered interchangeably. Structural diversity, glycosylation mode, and microbial biotransformation significantly affect cell uptake, target accessibility, and biological activity, resulting in significant differences between prototype ginsenosides, rare ginsenosides, microbial metabolites, and complex preparations.

Although the structure of ginseng extract is diverse, most of the ingredients are repeatedly concentrated in a relatively limited number of regulatory networks. Phosphatidylinositol 3-kinase (PI3K)/Akt, mitogen-activated protein kinase (MAPK), nuclear factor-κB (NF-κB), signal transducer and activator of transcription 3 (STAT3), mechanistic target of rapamycin (mTOR), oxidative stress signaling, epithelial-mesenchymal transition (EMT), and programmed cell death protein 1/programmed death-ligand 1 (PD-1/PD-L1) signaling pathways have been found in a variety of tumor types and experimental systems^22^, ^39^. Rather than representing independent mechanisms, these pathways function as regulatory hubs linking diverse hallmarks of cancer. Therefore, the biological activity of ginseng extract is best interpreted as the synergistic regulation of interrelated signal networks rather than the isolation of a single pathway. These comprehensive effects cover tumor cell survival, metastatic plasticity, tumor-immune interaction, metabolic adaptation, epigenetic regulation, and microbiome-related processes, as shown in **Fig. 3**.

### 3.1 Regulation of tumor-cell survival

The ability of malignant cells to maintain survival under continuous proliferative, metabolic, and therapeutic stress represents one of the defining characteristics of cancer^40^. Cell-cycle progression, mitochondrial integrity, oxidative homeostasis, and multiple forms of regulated cell death are tightly coordinated components of this adaptive program rather than independent biological events^41^. Current evidence indicates that ginseng-derived constituents interfere with several of these processes simultaneously, thereby reducing the capacity of tumor cells to maintain long-term viability.

Disruption of cell-cycle progression is one of the most consistently observed biological responses to structurally diverse ginseng-derived compounds. Although the underlying molecular mechanisms vary across tumor types, these compounds commonly restrict proliferative capacity by modulating cyclins, cyclin-dependent kinases, checkpoint proteins, and key regulators of mitotic progression. Representative examples include Rg1-mediated inhibition of Haspin-dependent histone H3 phosphorylation^42^ and SIRT1/TSC2 signaling^43^, Rb1-induced cell-cycle arrest accompanied by mitochondrial apoptosis^44^, ^45^, Rg3-mediated regulation of the circ_03074/miR-516b-5p/KPNA4 axis and of KIF20A expression and CDC25A proteasomal degradation^46^, ^47^. Comparable antiproliferative activity has also been described for Rh2^48^-^53^, Re/Rf^54^, ^55^, 20S-PPD^56^-^58^, and panaxadiol^59^, despite substantial structural differences among these compounds. Such consistency across multiple chemical scaffolds suggests that restriction of proliferative capacity represents a shared pharmacological characteristic rather than an isolated property of individual ginsenosides.

Limiting proliferation alone is insufficient to eliminate malignant cells, making induction of regulated cell death another major component of the anticancer response. Mitochondrial apoptosis remains the best-characterized mechanism, with repeated evidence demonstrating activation of caspases, disruption of mitochondrial membrane integrity, accumulation of ROS, and modulation of B-cell lymphoma 2 (Bcl-2) family proteins^60^, ^61^. Rare ginsenosides, particularly Rg3^62^-^66^ and Rh2^67^-^74^, consistently regulate these interconnected processes across diverse tumor models, whereas structurally related compounds including CK^75^-^77^ and several aglycones^78^-^80^ exhibit comparable pro-apoptotic activities through partially overlapping signaling pathways. However, Rg3 has also been reported to activate mTORC1 and promote cell growth under specific experimental conditions, indicating a context-dependent effect^81^. Although individual studies frequently emphasize distinct upstream regulators, most ultimately converge on restoration of mitochondrial susceptibility to programmed cell death.

Accumulating evidence further indicates that ginseng-derived constituents influence forms of regulated cell death extending beyond apoptosis. Autophagy has received the greatest attention, reflecting its dual role in supporting cellular adaptation under stress while also contributing to irreversible cell destruction under sustained perturbation. Depending on the biological context, Rh2^82^-^84^, Rg3^66^, Rk1^85^, and Rk3^86^, ^87^ regulate autophagic flux primarily through AMP-activated protein kinase (AMPK)/mTOR and PI3K/Akt-associated pathways. Ferroptosis has emerged as another promising mechanism, particularly in studies showing that CK induces FOXO-associated ferroptosis^88^. Direct evidence for pyroptosis remains insufficient. Current studies mainly indicate that CK^89^ and GPS^90^, ^91^ can regulate NLRP3-related inflammasome signaling, but whether this response culminates in canonical pyroptotic cell death requires further investigation.

In summary, these observations show that the main effect of ginseng extract is not to induce any single form of cell death. On the contrary, they destroy the interrelated regulatory network for tumor cells to survive under continuous biological stress. Therefore, cell cycle block, apoptosis, autophagy, iron death, and related reactions represent different biological manifestations of the same upstream disturbance, rather than independent pharmacological effects. This network perspective also explains why compounds with different structures repeatedly produce overlapping phenotypes even though they act on some different molecular pathways.

### 3.2 Regulation of tumor plasticity and metastatic progression

Metastatic dissemination represents one of the greatest obstacles to the success of cancer treatment and the main cause of cancer-related death. However, metastasis is no longer regarded as a simple sequence of tumor-cell detachment, migration, invasion, and colonization. On the contrary, it is now understood as a dynamic process driven by tumor plasticity. In the process, malignant cells constantly adjust their phenotypes through interaction with extracellular matrix components, matrix cell groups, vascular networks, and immune signals^92^. Therefore, therapeutic inhibition of metastasis not only needs to inhibit pathways related to migration, but also needs to destroy the adaptive procedures for tumor cells to acquire invasive, stem cell-like, and drug-resistant states.

Among these adaptive programs, EMT has been widely investigated in studies of ginseng-derived constituents^93^. Although EMT was initially described as a binary transition between epithelial and mesenchymal states, accumulating evidence indicates that tumor cells frequently occupy intermediate or hybrid phenotypes characterized by simultaneous epithelial stability and mesenchymal plasticity. These transitional states promote invasion, metastatic competence, cancer stemness^94^, ^95^, and can contribute to resistance to anticancer therapies^96^. Ginseng-derived compounds appear to restrict this phenotypic flexibility by restoring epithelial characteristics and reducing mesenchymal activation. Consistent changes in E-cadherin, N-cadherin, vimentin, Snail, and related regulators have been reported across multiple cancer models^97^, although these markers should be interpreted as indicators of phenotypic transition rather than definitive evidence of EMT reversal.

The anti-metastatic activity of ginseng-derived constituents involves coordinated regulation of several interconnected biological modules. Suppression of extracellular matrix remodeling represents one major mechanism, with repeated evidence showing reduced activity of matrix metalloproteinases (MMPs), particularly MMP-2 and MMP-9^98^-^104^, following treatment with multiple ginseng-derived compounds. These effects are frequently accompanied by inhibition of upstream signaling pathways involving PI3K/Akt, MAPK, Wnt/β-catenin, epidermal growth factor receptor (EGFR), transforming growth factor-β (TGF-β)/Smad, and STAT3^105^-^109^, all of which contribute to maintenance of invasive phenotypes. Rg3 provides one of the most extensively studied examples, demonstrating inhibition of EMT-associated programs across ovarian cancer^110^-^113^, osteosarcoma^108^, nasopharyngeal carcinoma^99^, ^114^, and colorectal cancer models^115^, ^116^. Importantly, the downstream mechanisms vary among tumor contexts, involving lncRNA-mediated regulation, β-catenin signaling, or EGFR-associated transcriptional control. These findings suggest that the biological outcome of EMT suppression is more conserved than the specific molecular route used to achieve it.

Beyond tumor-cell intrinsic regulation, ginseng-derived constituents also influence the surrounding tumor microenvironment. Rh2 and related compounds have been shown to modify cytokine networks^48^, ^117^-^119^, reduce pro-invasive signaling^120^, and regulate macrophage polarization^121^, ^122^, thereby limiting stromal support for tumor dissemination (**Table 2**). The ability to simultaneously affect malignant cells and their microenvironment may explain why certain rare ginsenosides produce stronger anti-metastatic phenotypes than prototype compounds. Rather than simply suppressing migration in isolated cell assays, these compounds appear to interfere with the reciprocal communication between tumor cells and stromal components that sustains metastatic progression.

Angiogenic remodeling represents another critical component of metastatic expansion. Tumor vascularization not only supplies nutrients and oxygen but also provides routes for systemic dissemination^123^, ^124^. Several ginseng-derived constituents inhibit angiogenic processes by reducing vascular endothelial growth factor (VEGF) signaling, endothelial-cell activation, microvessel formation, and vasculogenic mimicry^125^, ^126^. Among these, Rg3 has generated the strongest evidence, with anti-angiogenic activity demonstrated across endothelial models^127^, ^128^, zebrafish assays^129^, and xenograft systems^130^, ^131^. The reproducibility of these findings across experimental platforms suggests that interference with vascular adaptation may represent one of the more translationally relevant mechanisms associated with ginseng-derived compounds.

Despite extensive mechanistic research, there are still some important limitations. At present, most studies rely on wound healing experiments, Transwell invasive systems, or subcutaneous xenotransplant models, which can only reflect some of the characteristics of metastatic biology. There are still relatively few methods that are more physiologically relevant, such as spontaneous transfer models, in situ transplants, organoids of patient origin, and immunoactive systems. In addition, changes in EMT markers, MMP expression, or angiogenic factors may not prove the persistent inhibition of metastasis *in vivo*.

Taken together, the anti-metastatic activity of ginseng-derived constituents is best understood as the regulation of tumor plasticity, rather than inhibition of a single metastatic pathway. EMT, extracellular matrix remodeling, angiogenesis, and immune-tumor interactions are interrelated components in the adaptive procedures for the survival and proliferation of malignant cells. By limiting this plasticity, ginseng extract may reduce the ability of tumors to transition between proliferation, invasion, and drug resistance. Therefore, future research should go beyond static marker analysis and focus on building longitudinal, spatial, and clinically related models that can capture the transition evolution process.

### 3.3 Remodeling of tumor-immune interactions

The outcome of cancer progression depends not only on the intrinsic characteristics of malignant cells, but also on the balance between immune surveillance and immunosuppression in the tumor microenvironment. Effective antitumor immunity requires coordinated antigen identification, activation of cytotoxic immune cell groups, and maintenance of an immune microenvironment that can promote tumor clearance^132^. Cancer cells disrupt this balance through multiple strategies, including impaired antigen presentation, induction of immune checkpoints, secretion of immunosuppressive cytokines, and recruitment of regulatory immune populations^133^. Increasing evidence indicates that ginseng-derived constituents influence several components of this tumor-immune interaction network.

Immune checkpoint regulation is one of the most widely studied mechanisms. PD-L1 is the main mediator of tumor immune escape, and its expression is regulated by a variety of upstream pathways, including EGFR, STAT3, NF-κB, hypoxia-inducible factor 1-alpha (HIF-1α), and PI3K/Akt signaling. Some rare ginseng saponins can interfere with these regulatory loops, thus reducing the expression of PD-L1 or enhancing immunorecognition. Rg3 has attracted much attention because it can promote immunogenic tumor cell death^134^ and enhance T cell activation by regulating the stability and glycosylation of PD-L1^135^. Other compounds, including Rh4^136^, ^137^, Rk1^138^, Rh2^139^, and panaxadiol^59^, affect the expression of PD-L1 through different molecular mechanisms, indicating that checkpoint regulation is a convergence effect rather than compound specificity.

In addition to regulating immune checkpoints, ginseng extract can also directly affect immune effector function. Studies have reported that ginseng saponins Rg3, Rh2, and F1 can enhance the activity of natural killer (NK) cells by regulating activated receptors, stress response routes, and immunometric molecules^140^-^142^. Similarly, Rg1 can improve cytotoxic T cell response and enhance the efficacy of sequential immunotherapy^143^. These effects are particularly important because the direct tumor inhibition effect of ginseng saponins may complement the recovery of immune-mediated tumor removal function. Therefore, the anticancer activity of these compounds cannot be fully explained by the autonomic mechanism of tumor cells alone.

GPS represent a unique immunological category^144^, ^145^. Unlike many small-molecule ginsenosides, GPS usually has limited direct cytotoxicity but has a wider impact on immune cell communication. Its activity includes macrophage activation, dendritic cell maturation, NK cell stimulation, T cell response regulation, and inflammatory cytokine network regulation. This host-oriented mechanism of action may explain why GPS shows therapeutic potential when immune reconstruction is more important than direct tumor killing^146^-^149^. In addition, microbiota-dependent immune regulation further expands their biological relevance by linking intestinal microbial composition with systemic antitumor immunity^150^.

Existing evidence supports such a model: ginseng extract reshapes the immune microenvironment of tumors through a variety of complementary mechanisms. Small-molecule ginseng saponins mainly affect the signal transduction of immune checkpoints and the activation of immune effector cells, while GPS provide a wider range of immune support functions, involving innate and adaptive immunity. These unique and complementary activities provide a theoretical basis for the combined application of ginseng extract with modern anticancer therapies, including immunocheckpoint inhibitors, chemotherapy, and cell therapy.

However, the current evidence is still limited, mainly due to the lack of immune activity models and clinical verification. Many studies still rely on single-layer culture of tumor cells, which cannot reproduce complex immune interactions in patients. Future research should prioritize the construction of models containing the complete immune system, spatial immunospectroscopy analysis, and combined treatment strategies to determine whether ginseng extract can significantly improve the efficacy of existing immunotherapy.

### 3.4 Systems-level regulation: metabolic adaptation, epigenetic plasticity, and microbiome interactions

Cancer cells will constantly adapt to the fluctuations of nutritional supply, oxidative stress, immune stress, and therapeutic intervention. This adaptability is due to extensive metabolic remodeling^151^, epigenetic reprogramming^152^, and interactions with the host microbiome^153^, ^154^. Unlike mechanisms that directly regulate the proliferation or apoptosis of tumor cells, these processes affect the broader biological environment of tumor evolution. More and more evidence shows that ginseng extract can affect these systemic regulatory levels, thus providing a broader explanation for its diverse anticancer activity.

Metabolic reprogramming is one of the most consistently observed effects. Tumor cells usually rely on enhanced glycolysis, increased lactic acid production, and altered nutrient utilization to maintain rapid growth and survival under adverse conditions. Some rare ginseng saponins interfere with these metabolic adaptations by targeting key regulatory factors of glucose metabolism. For example, Rh2 inhibits glycolysis dependence in non-small cell lung cancer (NSCLC) by regulating the HIF-1α/PDK4 axis^71^ and STAT3/c-Myc signaling^155^, with associated regulation of glucose transport and glycolytic enzymes, including glucose transporter type 1 (GLUT1), pyruvate kinase isozyme type M2 (PKM2), and lactate dehydrogenase A (LDHA). Similar metabolic effects have been reported for Rg3 through regulation of glycolysis-associated signaling involving H19/miRNA networks^156^, STAT3/hexokinase 2 (HK2)^157^, and related metabolic regulators^158^. Instead of targeting a single metabolic enzyme, these compounds weaken the metabolic flexibility required for tumor cells to maintain growth under stress conditions.

In addition to glucose metabolism, ginseng-derived constituents also influence alternative nutrient pathways that support malignant progression. Rk1 suppresses glutamine utilization in hepatocellular carcinoma through inhibition of ERK/c-Myc signaling^159^, whereas CK reduces glutamine consumption and glutaminase activity in triple-negative breast cancer models^160^. Other derivatives, including 2-Deoxy-Rh2^161^, further demonstrate that structural modification can enhance metabolic interference by affecting both glycolytic activity and mitochondrial respiration. Related evidence from F2 also links ginseng-derived compounds to glycolytic regulation^162^. In short, these findings show that the regulation of metabolism by ginseng extract involves the synergistic disruption of interrelated energy-producing pathways, rather than simply suppressing the Warburg effect.

Epigenetic regulation provides another explanation for the extensive biological effects of ginseng extract. Tumor progression depends not only on genetic changes, but also on reversible changes in transcription processes that determine cell properties, plasticity, and therapeutic responses. Many studies have shown that ginseng extract can affect DNA methylation, histone-related regulation, RNA modification, and non-coding RNA networks. For example, 20S-Rg3 is associated with whole-genome methylation changes in breast cancer, including the regulation of KDM5A-related pathways^163^. Panaxatriol enhances METTL3-mediated N6-methyladenosine (m6A) modification of STUB1 and suppresses autophagy-related tumor cell survival^164^. It is reported that ginseng extract can also inhibit DNA methyltransferase activity and restore the expression of genes silenced by abnormal methylation in colorectal cancer models^165^. These findings show that ginseng extract may affect cancer biology by changing the regulatory mechanism that controls gene expression.

RNA-based epigenetic regulation has become another important mechanism, especially for Rh2 and its related compounds. Studies show that Rh2 is related to the regulation of long-chain non-coding RNA, microRNA network, and m6A modification pathway. Through these mechanisms, Rh2 affects transcription procedures related to immunomodulation^166^, tumor-cell proliferation^167^, ^168^, autophagy^169^, and stress adaptation. Although its precise molecular target is not fully clear, these findings expand the biological function of ginseng extract, making it beyond the traditional signaling pathway and suggesting that the regulation of cell identity may help it play an anticancer role.

Intestinal microbiota is another regulatory interface that connects the chemical composition of ginseng with the physiology of the host. Many ginseng saponins have limited oral bioavailability and undergo extensive microbial transformation in the intestine to produce metabolites with enhanced biological activity, including CK. On the contrary, ginseng-derived compounds can reshape the microbial community and change the microbial metabolites involved in inflammation, immunity, bile acid metabolism, and tumor development^170^. This two-way relationship distinguishes ginseng from many traditional anticancer drugs, because its biological activity is not only affected by direct interaction with tumor cells, but also by microbial processing and host-microbiota communication.

Emerging research provides more and more support for microbiome-related anticancer mechanisms. It is reported that Rh4 can inhibit the progression of colorectal cancer by regulating bile acid metabolism dependent on microbiota^171^. CK suppresses inflammation-associated colorectal tumorigenesis while simultaneously altering intestinal microbial composition^172^. GPS partially affects tumor development through microbiome remodeling and immune activation^173^. Rg1 has been associated with immune-cell recovery through modulation of the intestinal microbiota^174^, while Ro has shown microbiota-associated anti-liver-cancer activity^175^. Although most of these findings are still in the preclinical stage, they raise an important possibility that the composition of the microbiome may affect the efficacy and variability of ginseng-derived interventions.

In summary, metabolic adaptation, epigenetic plasticity, and microbiome regulation should not be regarded as independent mechanisms. On the contrary, they represent interrelated cancer regulatory levels and jointly determine how tumor cells respond to environmental pressure and treatment pressure. Ginseng-derived constituents seem to be able to influence this system-level network by regulating cell metabolism, transcription status, and communication between hosts and microorganisms. Future research on integrating metabolomics, epigenome, microbiome analysis, and pharmacokinetic analysis is crucial to clarify how these regulatory levels interact and how they can be applied to treatment.

### 3.5 Mechanistic convergence and evidence hierarchy

The preceding sections illustrate the remarkable diversity of biological effects attributed to ginseng-derived constituents. However, this diversity should not be interpreted as evidence of the infinite complexity of the mechanism. On the contrary, in different compound categories and tumor models, there is a consistent pattern: components with different structures repeatedly affect a relatively small number of regulatory networks, which coordinate a variety of cancer characteristics. Understanding this convergence is crucial to distinguish between true biological principles and observations that depend on specific situations.

At present, rare saponins provide the strongest evidence of mechanism in individual compounds. Compared with prototype saponins, the enhancement of their biological activity is usually due to differences in glycosylation, polarity, membrane interaction, and metabolic transformation^176^. Rg3, Rh2, and CK are the most in-depth examples of research, and their evidence covers tumor cell survival, metastatic plasticity, immunomodulation, metabolic adaptation, and microbiome-related effects. Therefore, these compounds are important models for understanding how structural modification affects pharmacological activity.

Prototype ginsenosides remain biologically relevant but generally display more moderate and context-dependent effects. Their importance may not be limited to direct anticancer activity, because they can act as precursors of microbial transformation and participate in the regulation of inflammation, immune response, and tumor-related signaling pathways. Chemical modification of derivatives and synthetic analogues further shows that structural optimization can change their effectiveness, selectivity, and action routes. However, activity enhancement does not necessarily mean an increase in therapeutic potential, because efficacy enhancement may be accompanied by reduced selectivity or adverse changes in pharmacokinetic properties.

GPS and complex preparations occupy a distinct pharmacological space. Comparative analyses of Panax GPS indicate that their structural heterogeneity is associated with diverse antitumor and immunoregulatory activities^177^. Their anticancer effects are less frequently associated with direct tumor-cell killing and more closely linked to immune regulation, macrophage activation, NK-cell function, and microbiome-mediated host responses. This difference highlights an important principle: different types of ginseng extracts may not compete with each other, but regulate complementary components in tumor biology. Small-molecule ginseng saponins may primarily affect the inherent vulnerability of tumor cells, while GPS may enhance the host-mediated antitumor defense mechanism.

Despite encouraging findings, the strength of mechanistic evidence varies considerably. Several pathways repeatedly appear in the literature, including PI3K/Akt, MAPK, NF-κB, STAT3, mTOR, EMT-associated regulators, PD-L1, VEGF, and MMPs. At the network level, several recurrent upstream-downstream relationships can be identified across different ginseng-derived constituents. Growth-factor and receptor-associated signals frequently converge on the EGFR/PI3K/Akt/mTOR axis, thereby supporting tumor-cell proliferation and survival and modulating autophagy in a context-dependent manner^178^, ^179^. Excessive oxidative stress and persistent endoplasmic reticulum stress can converge on mitochondrial dysfunction, loss of mitochondrial membrane potential, cytochrome c release, and subsequent caspase activation^180^. Hypoxia-associated HIF-1α/STAT3 cooperation regulates glycolytic and angiogenic transcriptional programs and can contribute to immune-effector dysfunction and resistance to PD-1 blockade^181^, ^182^. Meanwhile, TGF-β/Smad and Wnt/β-catenin-related transcriptional programs promote epithelial-mesenchymal plasticity, altered cell adhesion, invasion, and metastatic progression^183^, ^184^. At the host level, intestinal microbial transformation and bioconversion alter the formation and systemic exposure of less-glycosylated metabolites such as CK, thereby linking chemical structure and metabolism with pharmacokinetic behavior and potentially therapeutic efficacy^185^, ^186^. Together, these regulatory axes provide a mechanistic framework for interpreting the diverse phenotypes described in the preceding sections. However, alterations in these molecules should generally be interpreted as biological responses rather than definitive proof of primary target engagement. Many studies rely on pharmacological concentrations exceeding achievable systemic exposure, particularly for poorly absorbed ginsenosides^187^. In addition, rescue experiments, genetic validation, target occupancy studies, and pharmacokinetic correlation remain relatively uncommon.

A more rigorous evidence hierarchy is therefore required. Mechanisms supported by multiple experimental systems, genetic validation, and *in vivo* confirmation should be distinguished from observations based primarily on marker changes in cell culture. For example, the attenuation of Rh2-induced Bax translocation and apoptosis following voltage-dependent anion channel 1 (VDAC1) knockdown provides comparatively stronger evidence for a causal target-effect relationship than changes in protein expression alone^73^. Similarly, effects demonstrated in immunocompetent orthotopic models should carry greater translational weight than findings obtained from simplified xenograft systems. Establishing such a hierarchy will be critical for identifying which mechanisms represent genuine therapeutic opportunities and which reflect secondary cellular responses.

Overall, the current evidence supports a revised conceptual framework for understanding the anticancer activity of ginseng-derived constituents. These compounds should not be considered as collections of independent pathway inhibitors but rather as chemically diverse network modulators that influence interconnected regulatory systems governing tumor survival, plasticity, immunity, metabolism, and host interaction. This perspective reconciles the apparent mechanistic diversity of the literature and provides a foundation for future studies aimed at target validation, rational combination strategies, and clinical translation. The integrated mechanisms and evidence characteristics of representative ginseng-derived constituents are summarized in **Table 2**.

## 4. Ginseng-derived bioactive constituents: Multitarget regulators of tumor multidrug resistance and chemosensitization

Multidrug resistance (MDR) remains a major barrier to effective chemotherapy and is closely associated with treatment failure and tumor relapse^188^, ^189^. The molecular underpinnings of MDR are multifaceted, encompassing the overexpression of ATP-binding cassette (ABC) transporters such as P-glycoprotein (P-gp) and breast cancer resistance protein (BCRP), dysregulation of autophagy and apoptosis pathways, the influence of tumor-associated macrophages, and the pervasive immunosuppression within the TME^190^-^192^. Natural products have attracted attention in this setting because they can regulate multiple resistance-related pathways^193^, ^194^. Among them, ginseng-derived constituents have been investigated as chemosensitizing adjuvants that may interfere with resistance pathways while, in some settings, also mitigating treatment-related toxicity^195^.

A major mechanism involves regulation of drug efflux and compensatory survival pathways. Rh2 inhibits P-gp expression and restores paclitaxel sensitivity in resistant breast cancer models when incorporated into a nano-in-thermogel system^196^. Rg3 also affects transporter-related resistance. In breast cancer models, 20S-Rg3 combined with near-infrared photothermal therapy suppresses PI3K/Akt/mTOR signaling and reduces BCRP, P-gp, and multidrug resistance-associated protein 1 (MRP1) expression^197^. In lung cancer xenografts, 20S-Rg3 enhances cisplatin activity by downregulating P-gp, MRP1, and LRP1^198^. These findings suggest that ginsenosides may improve chemotherapy response partly by limiting drug efflux and weakening survival signaling.

Ginseng-derived compounds also regulate intracellular programs that support treatment resistance. In glioblastoma (GBM), 20S-Rg3 increases temozolomide sensitivity by suppressing MGMT, a DNA repair protein closely related to temozolomide resistance^199^. Rg1 reverses bortezomib resistance in multiple myeloma (MM) through AMPK/mTOR-mediated autophagy regulation^200^. 20S-PPT synergizes with EGFR tyrosine kinase inhibitors in resistant NSCLC by inhibiting EGFR phosphorylation and downstream signaling^201^. Panaxynol targets Hsp90 and suppresses acquired chemoresistance in NSCLC^202^.

Resistance can also be shaped by immune escape, metabolic adaptation, and extracellular matrix remodeling. 20R-Rg3 counteracts chemotherapy-induced PD-L1 upregulation by inhibiting NF-κB p65 and Akt signaling, thereby restoring T-cell cytotoxicity in NSCLC models^203^. In tamoxifen-resistant breast cancer, 20S-Rg3 targets 6-phosphofructo-2-kinase/fructose-2,6-biphosphatase 3 (PFKFB3) and interferes with glycolytic adaptation^204^. In pancreatic ductal adenocarcinoma, Rh2 reverses gemcitabine resistance by downregulating laminin subunit gamma 2 (LAMC2), linking extracellular matrix remodeling with chemoresistance^205^. These findings extend the anti-MDR profile of ginsenosides beyond classical transporter inhibition. The expected benefits may vary depending on the treatment method. In combined chemotherapy treatment, ginseng extract may restore intracellular drug exposure and apoptosis ability; while in immunotherapy-oriented combined treatment, its potential value is mainly reflected in reducing PD-L1-mediated immune escape or improving effector cell function. However, at present, most of the evidence of combined treatment is still in the preclinical stage, and the optimal dose, treatment sequence, pharmacokinetic interaction, and additional toxicity of these combined treatments have not been fully clarified.

Current evidence suggests that ginseng-derived constituents may improve anticancer efficacy through coordinated effects on drug efflux, apoptosis, autophagy, DNA repair, metabolic adaptation, and immune-or matrix-associated resistance. Among them, Rg3 and Rh2 are the most frequently studied compounds in MDR reversal and chemosensitization. Some sensitizing effects, however, are observed in specific resistant cell models, combination systems, or nanoformulations, and should therefore be interpreted in relation to drug exposure, formulation design, and tumor context.

## 5. Clinical translation and advanced delivery strategies for ginseng in oncology

The integration of ginseng-derived preparations into modern oncology has been explored mainly through adjunctive clinical use and pharmaceutical formulation development. Clinical translation has progressed particularly in China, where ginsenoside-containing preparations such as Shenyi Capsule have been used as adjunctive agents in cancer treatment^206^. Furthermore, ginseng extracts are integral components of widely used composite formulations. Aidi injection, a composite formulation containing ginseng, has shown mitochondria-associated pro-apoptotic activity in experimental hepatocellular carcinoma (HCC) models^207^. Similarly, GPS injections have been evaluated as adjunctive preparations for alleviating treatment-associated immune suppression and reducing radiotherapy-or chemotherapy-related adverse effects^147^. However, most available evidence still comes from domestic studies, systematic reviews, meta-analyses, or health technology assessments. Small sample sizes, heterogeneous regimens, variable endpoints, and insufficient high-quality multicenter randomized trials limit the overall strength of evidence. Therefore, these preparations should currently be regarded as adjunctive or supportive agents rather than alternatives to standard anticancer therapy.

In terms of guideline positioning, ginseng-related preparations are more commonly used in Chinese integrative oncology practice, whereas their recognition in international oncology guidelines remains limited. Notably, recent ASCO-SIO guidance mentions American ginseng mainly for cancer-related fatigue, rather than positioning ginseng-derived preparations as standard antitumor agents^208^. This gap highlights the need for stronger clinical validation, standardized preparations, and clearer indications.

At the same time, it should be considered in combination with the pharmacological activity summarized in Section 3 and the pharmacokinetic behavior of each ginseng saponin. Rg3 is a typical example: although it can regulate cell apoptosis, angiogenesis, immune response, and drug resistance, its poor water solubility, low membrane permeability, and low bioavailability limit its drug development and systemic exposure^209^. Rh2 is affected by poor solubility, intestinal metabolism, and P-gp-related efflux^210^, and CK is constrained by poor aqueous solubility, insufficient absorption, and limited and variable tissue exposure^211^. Therefore, drug delivery systems (DDS) should match the main obstacles of each active ingredient, and not just regard it as a general-purpose nanotechnology.

Compared with traditional methods such as oral mixed suspension, simple injection, or non-targeted preparations, advanced DDS provide more targeted solutions for improving bioavailability, stability, controlled release, and tumor accumulation. Liposomes and precursor liposomes are especially suitable for improving the dispersion and oral bioavailability of ginseng saponin Rg3^212^. Polymer- or albumin-based nanoparticles are more suitable when the main goal is to protect unstable compounds, achieve surface ligand-mediated targeting, prolong residence time, or combine delivery with chemotherapy drugs^213^. Ginsenoside-based liposomes represent a further step, because amphiphilic ginsenosides such as Rg3 and Rh2 may function not only as therapeutic cargos but also as bioactive membrane components, thereby linking delivery improvement with intrinsic pharmacological activity^214^. Polymer micelle and emulsion systems are mainly suitable for hydrophobic or orally restricted compounds, such as CK, Rh1, and Rh2. The solubility, intestinal absorption, CYP450 metabolism, and P-gp excretion of these compounds are the main concerns. The main delivery platforms are illustrated in **Fig. 4**, while the compound-specific relationships between pharmacokinetic barriers and delivery strategies are summarized in** Table 3**.

### 5.1 Nanoparticle-based platforms for enhanced targeting and efficacy

Nanoplatforms provide a flexible strategy for matching the physicochemical and pharmacokinetic limitations of ginseng-derived compounds with specific delivery designs. For poorly soluble ginsenosides, nanocarriers can improve dispersion and apparent solubility. For unstable or rapidly cleared compounds, they may protect the payload and prolong systemic exposure. For compounds requiring tumor-selective delivery, surface modification, biomimetic coating, or ligand-mediated targeting can enhance tumor accumulation and cellular uptake^213^.

Polymeric, albumin-based, and biomimetic nanoparticles (NPs) mainly address poor solubility, insufficient stability, limited tumor accumulation, and the need for co-delivery. Rg3-loaded functionalized graphene oxide (PEG-GO-FA/ICG-Rg3)^215^ and tumor-cell-membrane-coated PLGA NPs (Rg3-PLGA@TMV) have been used to improve tumor targeting, controlled release, immune activation, or co-delivery with doxorubicin^216^. PEGylated ginsenoside conjugates, including PEG-Rh1/Rh2 and PEG-PPD, improve water solubility, circulation behavior, and antitumor performance^217^, ^218^. Other representative systems, including an Rh2-loaded nanoniosomal formulation^219^, BSA-Rh2 NPs^220^, and folic acid-targeted Rg5 albumin NPs^221^, further support the value of protein-or vesicle-like carriers for increasing cellular uptake and tumor selectivity.

Several self-assembled or composite nanoparticle systems also connect delivery improvement with biological activity. Dox@Rg1 NPs enhance the antitumor activity of doxorubicin while reducing cardiotoxicity^222^, and Rb1-stabilized PTX/PPD co-loaded NPs (GPP NPs) improve tumor targeting and TME modulation^223^. Mesoporous silica NPs incorporating ginseng-derived components (ICG/CMSN@GsE) increase drug retention and macrophage-related targeting^224^, while CK-loaded carboxymethyl chitosan calcium NPs (CK-OCMC NPs) improve CK uptake and caspase activation in PCa cells^225^. These examples suggest that nanoparticle design can be used not only to improve drug exposure, but also to integrate ginseng-derived compounds with chemotherapy, phototherapy, or TME-directed strategies.

Metallic nanoparticles and carbon-based systems provide additional delivery or imaging-related possibilities, although their translational profile requires careful evaluation. CK-loaded gold NPs prepared using *Lactobacillus kimchicus* DCY51T enhance CK-related apoptosis and may support photothermal therapy^226^, while the gold and silver GBAu NPs and GBAg NPs, mediated by ginseng berry, have shown antioxidant, antimicrobial, and cytotoxic activities in experimental models^227^. Silver nanoparticles derived from ginseng root^228^, or fresh leaf extracts (P.g AgNPs)^229^, as well as ginseng-root-derived zinc oxide NPs^230^, have been associated with cytotoxicity, EGFR-related inhibition, or mitochondria-mediated apoptosis in cancer models. Carbon dots (CDs) and carbon nanotubes (CNTs) also broaden this field. Ginsenoside-based CDs^231^, ^232^ and ginseng-extract-derived CDs^233^ show antitumor or immune-related activity, whereas CNT-based formulations carrying Rb1/Rg1^234^ or Rb3^235^ have enhanced anti-proliferation effects in the experimental model. Compared with polymer or lipid matrix systems, these platforms may provide additional optical, photothermal, or structural advantages, but their long-term safety, biodegradation, and manufacturability need stricter assessment.

Unlike inert carriers, ginseng-derived nanoparticles (GDNPs) and ginseng-derived exosome-like nanoparticles (GENs) contain natural lipids, proteins, and other bioactive components that may contribute directly to their biological effects. GDNPs have been reported to inhibit EMT through pentose phosphate pathway regulation^93^, reprogram macrophages to alleviate T-cell exhaustion^236^, and convert immunosuppressive tumors toward a more T-cell-inflamed phenotype^237^. Other studies show that GDNPs promote M2-to-M1 macrophage polarization and induce ROS-related apoptosis^238^. In preclinical models, GENs have been reported to cross the blood-brain barrier, accumulate in glioma, and modulate tumor-associated macrophages^239^. These vesicle-like systems are particularly related to TME remodeling and immunomodulation, but their component definition, batch consistency, and quality control are still the core issues for further development.

### 5.2 Liposomes: biomimetic and self-assembling systems

Liposomes are phospholipid bilayer vesicles that can encapsulate poorly soluble compounds, improve dispersion, protect unstable payloads, and reduce nonspecific toxicity. Surface modification can further enhance the targeting of tumors. In the ginseng-related delivery system, liposomes are particularly attractive because amphiphilic ginseng saponins (such as Rh2) can partially replace cholesterol-like stabilizing components and directly participate in membrane assembly. This forms a "carrier-drug" design, in which ginseng saponins not only contribute to drug delivery, but also have therapeutic activity^214^.

Rg3-based liposomes provide representative examples of this strategy. Folate-modified Rg3 liposomes improve solubility and targeting in TNBC models^240^. Rg3-PTX liposomes (Rg3-PTX-LPS) deliver paclitaxel and remodel the TME by promoting M2-to-M1 macrophage repolarization through IL-6/STAT3-related regulation^241^. Other Rg3-based liposomal systems have been applied to glioma and TNBC, including Rg3-based glioma liposomes^242^ and gambogic-acid-loaded biomimetic liposomes (G/R-MLP)^243^, which enhance tumor targeting, migration inhibition, and therapeutic efficacy. These systems are particularly useful for combinations where cytotoxic delivery and TME modulation are both desired.

Liposomes based on ginseng saponin Rh2 further clarify the structural value of ginseng saponins in lipid carriers. PTX-Rh2 liposomes (PTX-Rh2-lipo)^244^, LTL@Rh2@Lipo-GE11^245^, and ACG-loaded Rh2 liposomes (ACGs+Rh2)-Lipo^246^ containing ginseng saponin ACG have improved Drug delivery efficiency and tumor targeting in lung cancer, breast cancer, and glioma models. Compared with traditional cholesterol- or PEG-based liposomes, liposomes containing ginseng saponin Rh2 may have higher membrane stability and inherent antitumor activity. Mixed liposomes containing Rh2 and other natural products also showed enhanced anti-lung cancer effects^247^. In addition, TPGS-modified CK liposomes (GCKT-Lipo) improve the water solubility and antitumor efficacy of CK, and support the use of liposomes to treat poorly soluble microbial metabolites^248^.

Therefore, the liposomal system is most suitable for ginseng derivatives that require increased solubility, membrane stabilization, tumor targeting, or combined administration. Compared with simple encapsulation, ginseng-based liposomes have the additional advantage of combining carrier function with pharmacological activity, especially suitable for Rg3, Rh2, and CK.

### 5.3 Polymeric micelles for improved bioavailability

Polymeric micelles are self-assembled nanoscale carriers formed from amphiphilic polymers in aqueous solution. Their hydrophobic core can encapsulate poorly soluble compounds, while the hydrophilic shell improves dispersion, reduces premature clearance, and prolongs circulation time^249^. These features make micelles particularly suitable for hydrophobic ginsenosides or metabolites whose application is limited by poor aqueous solubility and low oral bioavailability.

CK and Rh2 are representative examples. CK has broad anticancer activity, but its poor solubility and limited bioavailability restrict its *in vivo* performance. A54 peptide-modified deoxycholic acid-O-carboxymethyl chitosan micelles loaded with CK (APD-CK) improve liver targeting and antitumor activity^250^. Rh2 mixed micelles (Rh2-M) prepared by thin-film dispersion enhance cellular uptake, tumor targeting, and anticancer activity^251^. These studies show that micellar systems can improve the pharmaceutical performance of hydrophobic ginsenosides without requiring covalent structural modification.

Polymeric micelles are most suitable as exposure enhancement systems for compounds such as CK and Rh2. Their main value is to improve solubility, whole-body exposure, and tissue targeting. Compared with more complex multifunctional nanoparticles, micelles may provide a simpler preparation strategy, but stability, drug delivery efficiency, release behavior, and repeatability of amplified production still need to be considered.

### 5.4 Nanoemulsion-and microemulsion-based systems

Nanoemulsions and microemulsions are colloidal delivery systems composed of an oil phase, aqueous phase, surfactant, or cosurfactant. Microemulsions are thermodynamically stable, whereas nanoemulsions are kinetically stable and generally require external energy for their preparation. They are useful for improving solubilization, oral absorption, and combination delivery of lipophilic compounds^252^. Unlike polymeric nanoparticles or liposomes, emulsion-based systems are particularly suitable for oral or multicomponent formulations in which solubilization and intestinal absorption are the main barriers.

This strategy is relevant to Rh1 and Rh2, whose oral bioavailability is limited by intestinal CYP450-mediated metabolism and P-gp efflux^253^. A multicomponent microemulsion containing etoposide, coix seed oil, and Rh2 (ECG-MEs) improved the antitumor efficacy of Rh2 in A549 xenograft models, with increased apoptosis and tumor growth inhibition^254^. Another example is a self-microemulsifying delivery system co-loading Rg3, *ganoderma lucidum* polysaccharide (GLP), and oridonin (RGO-SMEDDS). This system was reported to reduce immunosuppressive cytokine production and M2-like macrophage polarization, inhibit VEGF-related angiogenesis, and suppress EGFR/Akt/GSK-3β signaling in HCC models^255^.

The emulsion system is popular because it can improve solubility, oral absorption rate, and combined administration ability at the same time. As for ginseng extracts, the emulsion system is especially suitable for multi-component preparations aimed at enhancing drug exposure and integrating immunomodulatory or anti-angiogenic effects. Its transformation application potential will depend on the further optimization of the robustness, stability, and repeatability of the preparation.

## 6. Discussion and future perspectives

More and more experimental evidence shows that ginseng extract has a wide range of anticancer potential, which can be achieved by coordinating and regulating tumor cell survival, metastatic behavior, immune response, metabolic adaptation, epigenetic status, and therapeutic drug resistance. However, the future development of ginseng-based oncology should move beyond the accumulation of mechanistic observations and toward a more integrated framework that connects chemical identity, molecular mechanism, pharmacological exposure, and clinical outcome. Therefore, the core challenge is not only to discover new anticancer active ingredients, but also to determine which ingredients can produce repeatable effects, through which causal mechanisms, and under what treatment conditions these effects can be transformed into clinical benefits. For immunotherapy-oriented combined therapy, this transformation should also take into account the existing challenges in biomarker selection, model correlation, joint program design, and clinical implementation^256^.

The main premise of this transformation is to improve the definition and standardization of chemical components. The biological activity of ginseng products is significantly affected by the species source, cultivation environment, processing technology, extraction method, and preparation strategy. This variability is an important reason for the inconsistency of experimental results and complicates the comparison between different studies. For ginseng saponins and GPS, this problem is particularly prominent because their chemical composition will change due to steaming, fermentation, intestinal microbial transformation, or structural modification^170^, ^176^. Therefore, future research should combine comprehensive chemical characterization with pharmacological evaluation, including standardized preparation methods, quantitative analysis of biologically active ingredients, and multi-component quality control methods^257^. The establishment of a reliable chemical framework will lay the foundation for repeatable mechanism research and reasonable clinical development.

Beyond chemical standardization, a deeper understanding of molecular mechanisms is required. The multitarget nature of ginseng-derived compounds may represent an intrinsic advantage in cancer biology because malignant progression involves interconnected processes rather than isolated pathways. Nevertheless, at present, many studies are still mainly at the descriptive level, only reporting changes in signaling molecules such as PI3K/Akt, MAPK, NF-κB, STAT3, mTOR, and autophagy-related pathways, without fully clarifying their causal contribution to treatment results^258^. Future research should therefore shift from pathway cataloging toward mechanistic validation by incorporating genetic manipulation, rescue experiments, target identification approaches, multi-omics integration, and clinically relevant models such as patient-derived organoids and xenografts. Such strategies will help distinguish between the main molecular targets and downstream reactions, and improve the rational design of ginseng-derived therapies.

Successful clinical transformation also depends on overcoming pharmacological limitations. Many bioactive ginseng saponins show strong anticancer activity in experimental systems, but due to poor solubility, limited absorption, rapid metabolism, or insufficient tumor accumulation, whole-body exposure is insufficient. Advanced delivery strategies provide promising solutions by improving pharmacokinetic behavior and achieving more accurate interaction with tumor tissue. However, the future development of DDS should not focus solely on increasing drug concentration. On the contrary, the design of the preparation should be combined with the biological mechanism to determine how, where, and when the ginseng-derived molecules work. Issues such as production scale, preparation stability, long-term safety, metabolic pathways, and the potential impact of therapeutic drug resistance are still key issues that need to be systematically evaluated^259^-^261^.

Emerging technologies may provide new opportunities to redefine the therapeutic potential of ginseng-derived compounds. Tumor microenvironment-responsive delivery systems, biomimetic nanoparticles, and exosome-based platforms may enable controlled release and improved combination strategies with chemotherapy or immunotherapy^259^. At the mechanistic level, unexplored areas such as mitochondrial epigenetic regulation, mitochondrial DNA-related signal transmission, and metabolic-epigenetic interaction may reveal other dimensions of ginseng-mediated cancer regulation^262^, ^263^. At the same time, the progress of high-throughput sequencing, spatial multi-omics, artificial intelligence-based drug discovery, and computational screening technology may accelerate the identification of key targets and optimize the development of saponin derivatives^264^, ^265^.

Ultimately, the next generation of ginseng oncology research requires a closer integration of natural product chemistry, molecular oncology, pharmacology, and clinical medicine. Mechanism research should give priority to the identification of causal targets, not pathway association. Drug research and development should regard delivery optimization as an important part of treatment plan design, rather than post-remedial measures. Clinical research should focus on clear indications, reasonable combined drug strategies, pharmacological administration schemes, and clinically significant efficacy indicators. Through this integrated framework, ginseng-derived drugs are expected to gradually develop from empirical auxiliary products to mechanism-oriented and evidence-based tumor treatments.

## 7. Conclusion

Ginseng-derived compounds represent a unique class of natural product-based candidates with diverse but interconnected anticancer activities. More and more evidence shows that ginseng saponins, GPS, and their related derivatives regulate cancer progression through synergy in tumor cell survival, metastatic plasticity, immunoregulation, metabolic adaptation, epigenetic regulation, and therapeutic drug resistance. These compounds do not act as traditional cytotoxic drugs, but seem to affect multiple biological levels, together shaping tumor behavior and therapeutic response. Among the most extensively investigated constituents, Rg3, Rh2, and CK provide the strongest mechanistic evidence, but their relative therapeutic value still depends on drug exposure, tumor microenvironment, and clinical verification.

Although remarkable progress has been made in the experiment, ginseng-derived agents remain in an intermediate stage between mechanistic discovery and clinical application. Their most realistic applications in the near future may be in adjuvant treatment, enhancing therapeutic effects, regulating immunity or metabolism, and combined treatment strategies, rather than replacing existing anticancer therapies. Advances in drug delivery technology, chemical standardization, and pharmacokinetic optimization may help overcome the current limitations in bioavailability, target exposure, and preparation consistency.

Future progress will require an integrated development framework that links chemical characterization, causal mechanism verification, transformational pharmacology, and clinical evaluation. Identifying active ingredients, identifying clinically relevant targets, establishing exposure-reaction relationships, and carrying out rigorously designed clinical trials are crucial to determine where ginseng extracts play a significant therapeutic role. Through such mechanism-guided approaches, ginseng may gradually evolve from a traditional medicinal resource into a scientifically defined and clinically relevant component of modern oncology.

## Figures and Tables

**Figure 1:**
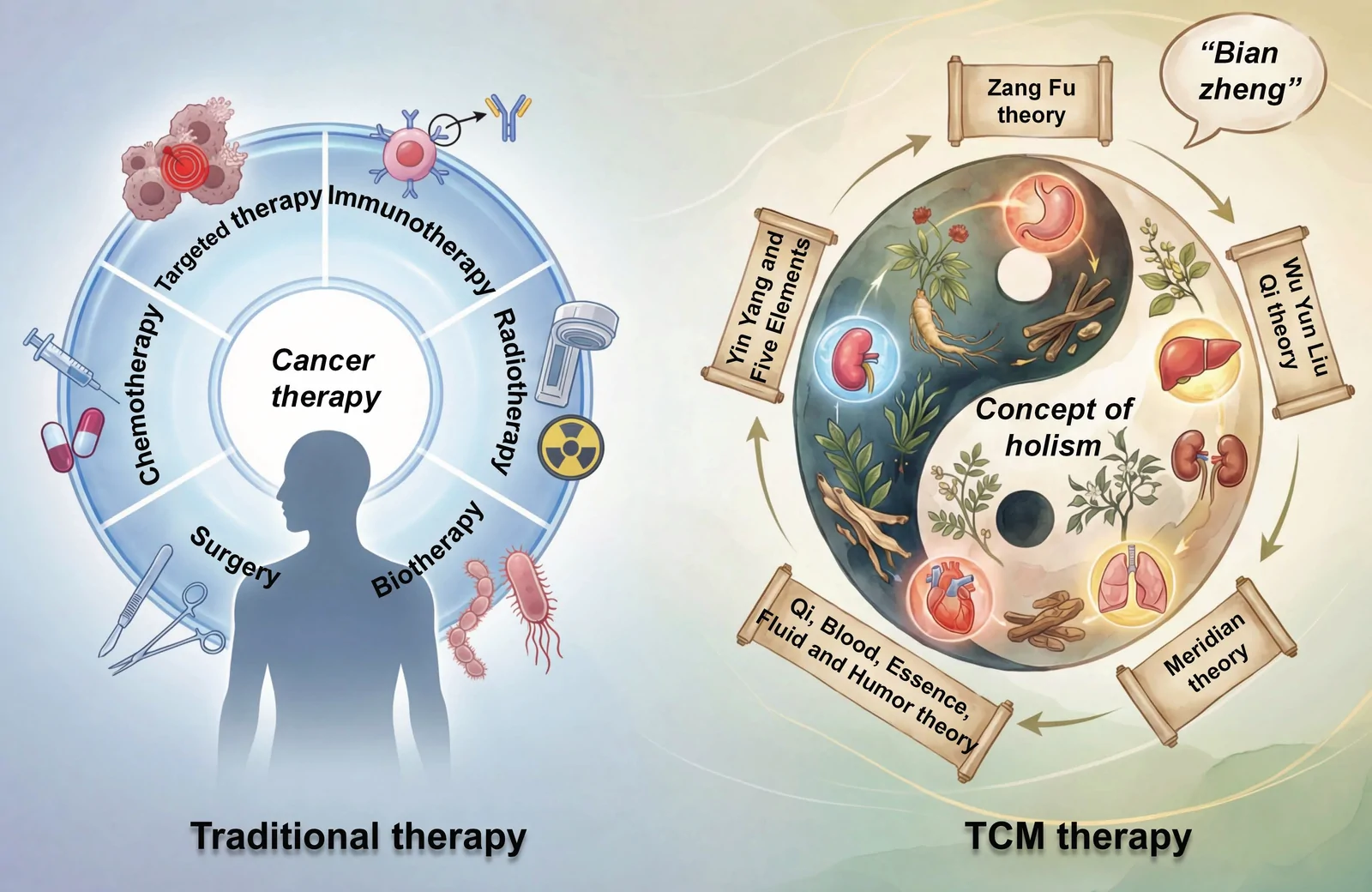
Comparison between traditional and TCM treatments for cancer.

**Figure 2:**
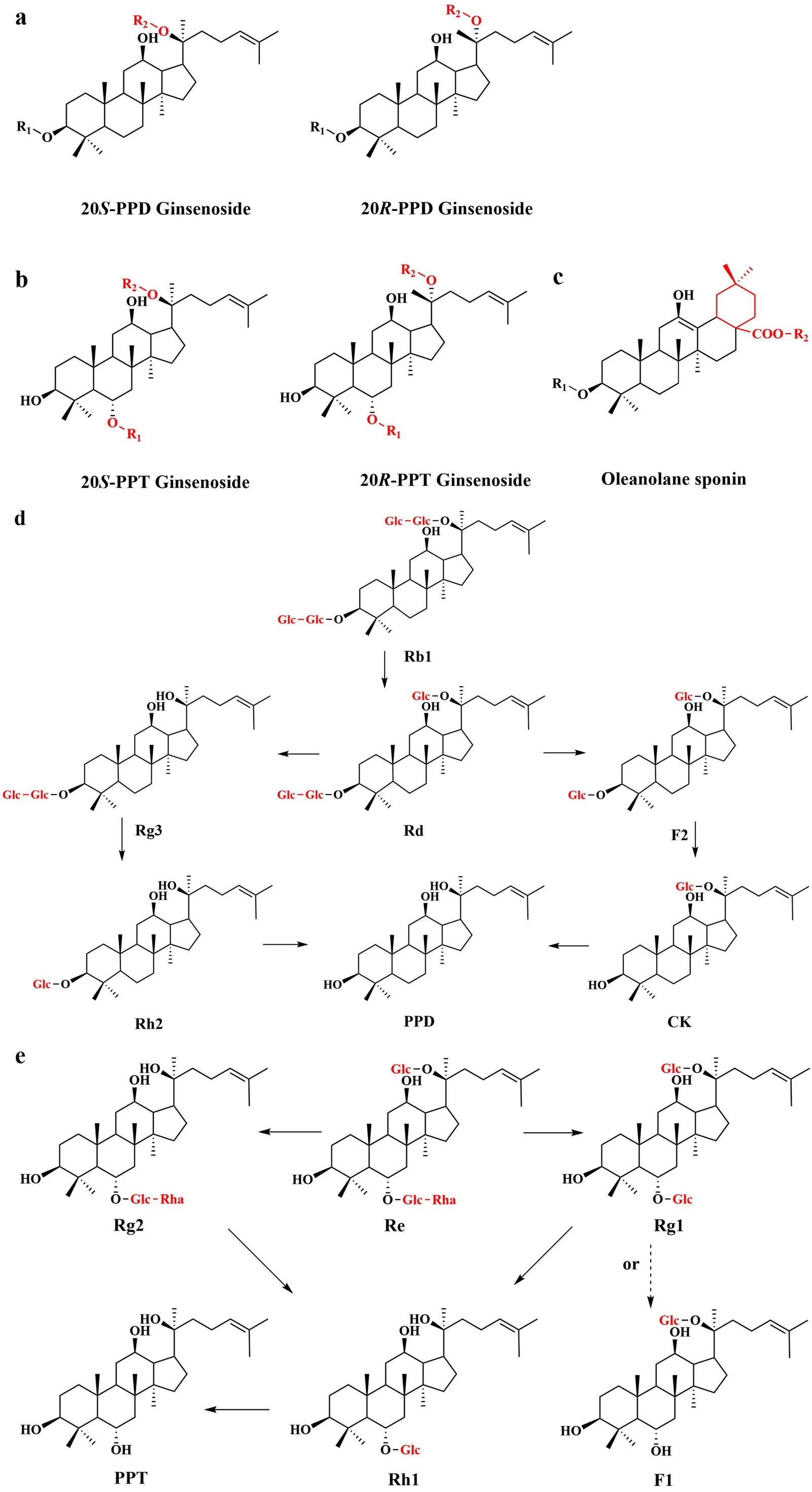
The scaffold structure of ginsenosides and the interconversion between saponins. a. 20S-Protopanaxadiol (PPD)-type saponins and 20R-Protopanaxadiol (PPD)-type saponins; b. 20S-Protopanaxatriol (PPT)-type saponins and 20R-Protopanaxatriol (PPT)-type saponins; c. Oleanolic acid (OA); d. Major metabolic processes of PPD-type ginsenosides; e. Major metabolic processes of PPT-type ginsenosides.

**Figure 3:**
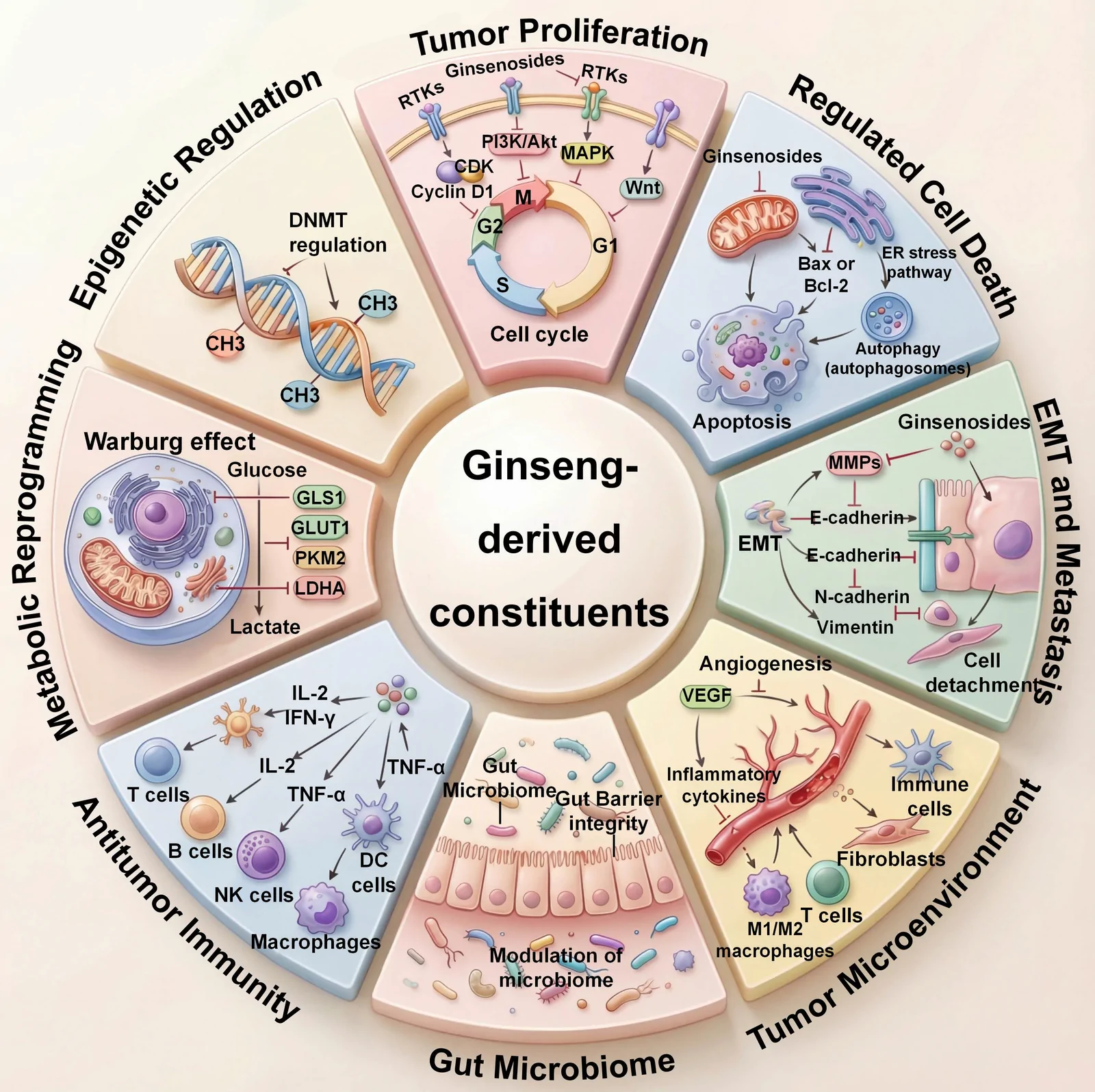
Multi-target anticancer mechanisms of ginseng-derived constituents in tumor regulation. Ginseng-derived constituents exert pleiotropic anticancer effects by regulating tumor cells, immune components, metabolism, and the tumor microenvironment. These compounds inhibit tumor proliferation through modulation of PI3K/Akt/mTOR, MAPK, Wnt, and cell-cycle signaling pathways; induce apoptosis via reactive oxygen species (ROS)-mediated mitochondrial dysfunction and caspase-dependent pathways; and suppress epithelial-mesenchymal transition (EMT), angiogenesis, and metastasis through regulation of matrix metalloproteinases (MMPs), vascular endothelial growth factor (VEGF), and adhesion molecules. Furthermore, ginseng compounds remodel antitumor immunity by activating immune effector cells and regulating inflammatory cytokines, while affecting tumor metabolism through inhibition of the Warburg effect and glycolytic reprogramming. Their regulation of intestinal flora further promotes systemic anticancer activity and highlights the multidimensional therapeutic potential of ginseng extract.

**Figure 4:**
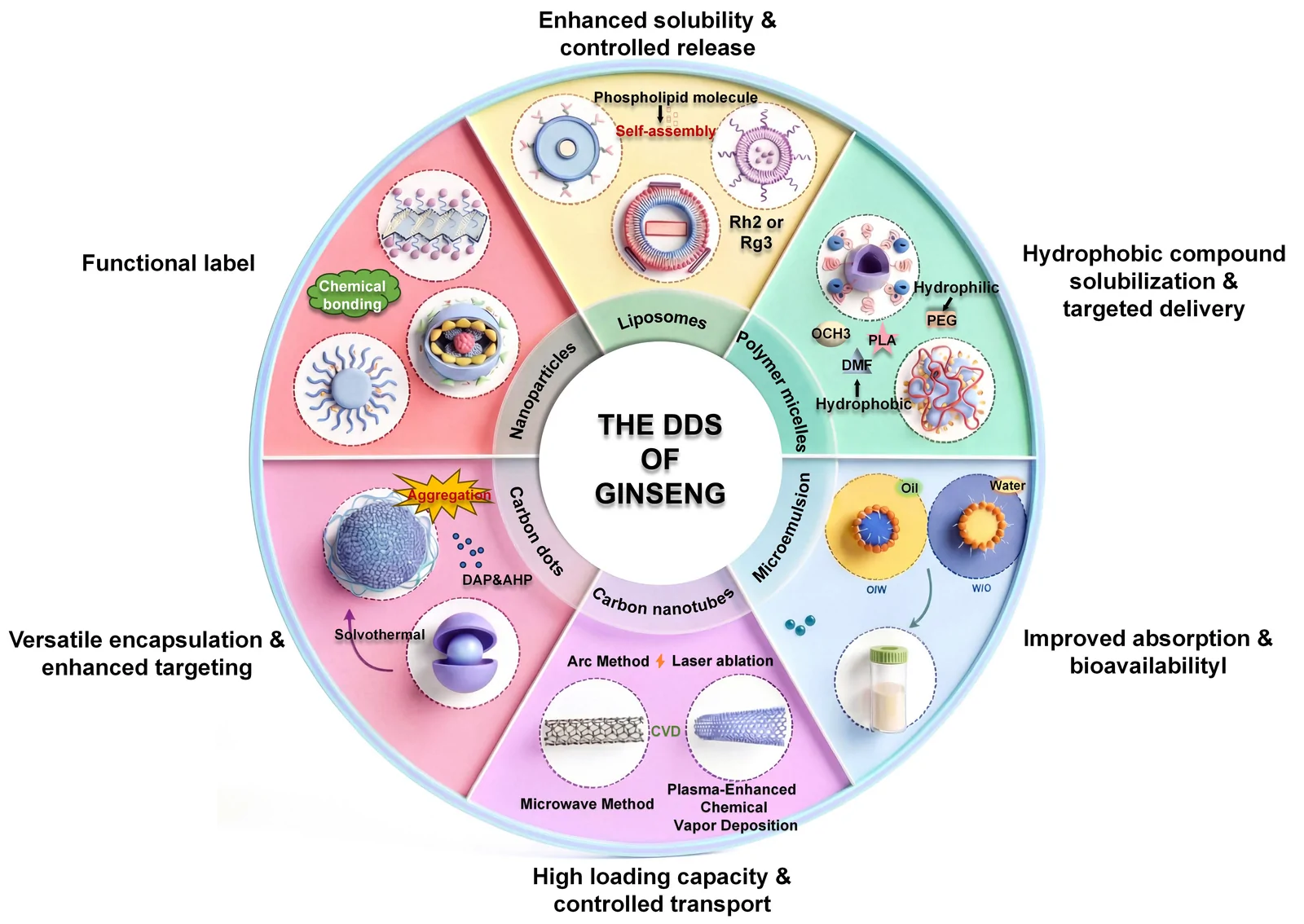
Nanotechnology-based delivery strategies for enhancing the bioactivity of ginseng-derived compounds. Ginseng-derived bioactive compounds, especially ginseng saponins Rg3 and Rh2, have a variety of biological activities, but their applications are limited by poor water solubility, low bioavailability, and insufficient delivery efficiency. Nanotechnology-based drug delivery systems (nano-DDS), including liposomes, polymer micelles, nanoemulsions, nanoparticles, and carbon-based nanocarriers, have been developed to improve the pharmacological properties of these natural compounds. Through packaging, self-assembly, and chemical modification strategies, these nano-platforms can improve the stability, solubility, controlled release, and targeted delivery ability of compounds, thus enhancing the therapeutic potential of bioactive compounds derived from ginseng. These advances provide a promising way to transform ginseng ingredients into effective biomedical applications.

**Table 1:** Representative classification of major ginsenosides, metabolites, and aglycones

Classification	Prototype ginsenosides	Rare ginsenosides and metabolites
PPD	Ginsenoside Rb1, Rb2, Rc, Rd	Ginsenoside Rg3, Rg5, Rh2, Rk1, F2, CK, aPPD
PPT	Ginsenoside Rg1, Rf, Re	Ginsenoside Rh1, Rh4, Rg2, Rk3, F1, aPPT
OA	Ginsenoside Ro	Not listed

**Note:** Ginsenosides are classified according to their aglycone skeletons into protopanaxadiol (PPD)-, protopanaxatriol (PPT)-, and oleanolic acid (OA)-type compounds. Prototype ginsenosides are naturally abundant parent saponins, whereas rare ginsenosides and metabolites are generally present at low levels or are generated through processing, deglycosylation, or intestinal microbial biotransformation. CK, compound K; aPPD, protopanaxadiol aglycone; aPPT, protopanaxatriol aglycone. “Not listed” indicates that no representative rare OA-type compound was included in this summary table.

**Table 2:** Integrated anticancer mechanisms and evidence profiles of representative ginseng-derived constituents

Representative constituent	Anticancer process	Key molecular pathways or mechanisms	Representative experimental evidence and evidence status	Major references
Ginsenoside aglycones 20S-PPD, 20S-PPT, panaxadiol, and panaxatriol	Tumor growth and regulated cell death	Cell-cycle arrest; PI3K/Akt/mTOR and HIF-1α/STAT3 inhibition; mitochondrial/caspase-dependent apoptosis; ROS/ER stress and autophagy	*In vitro*: several tumor-cell models. *In vivo*: selected xenograft models. Status: predominantly preclinical; evidence is concentrated in a limited number of aglycones	58, 59, 79, 80, 266
	Metastasis, immune escape, and epigenetic regulation	20S-PPD: SIRT1- and EGFR/MAPK-related EMT and MMP-2/9 inhibition; panaxadiol: HIF-1α/STAT3/PD-L1 suppression; panaxatriol: METTL3/m6A-STUB1 regulation	*In vitro*: EMT, invasion, checkpoint, and epigenetic assays. *In vivo*: selected metastasis models. Status: immune and epigenetic evidence remains limited	59, 105, 164, 267
Rg1	Cell-cycle regulation, metastasis, immunity, and microbiome modulation	Haspin/histone H3 and SIRT1/TSC2 regulation; TGF-β1/EMT and MMP-2/9 inhibition; enhanced T-cell function and macrophage/dendritic-cell regulation; gut microbiota remodeling	***In vitro*:** cancer-cell and immune-cell studies. ***In vivo*:** adoptive immunotherapy and microbiota-related animal models. **Status:** multidomain but context-dependent; direct cancer-specific microbiome evidence is limited	42, 43, 98, 118, 143, 174, 268
Rb1	Tumor growth, apoptosis, TME inflammation, and angiogenesis	p53/Bax/cytochrome c/caspase-mediated mitochondrial apoptosis; hypoxia-induced EMT inhibition; reduced TNF-α and IL-6; miR-33a/PEDF/PPAR-γ-mediated antiangiogenesis	***In vitro*:** tumor-cell and endothelial models. ***In vivo*:** cancer-cachexia and angiogenesis-related models. **Status:** evidence is distributed across a relatively small number of models	44, 45, 119, 126, 269
Rd	Tumor growth, apoptosis, EMT, and stemness	G0/G1 arrest; p53/Bax- and caspase-mediated apoptosis; H19/miR-675-5p/CDH1, miR-18a/Smad2, and EGFR/Akt-related EMT regulation	***In vitro*:** lung, gastric, tongue, breast, and colorectal cancer models. ***In vivo*:** selected xenografts; some human-tissue association. **Status:** moderate preclinical support without clinical intervention evidence	106, 270-274
Rb2	EMT, metastasis, and cancer stemness	Suppression of EGFR/Akt/SOX2 and TGF-β/Smad signaling; reduced EMT, MMP activity, and stemness-associated genes	***In vitro*:** colorectal cancer migration, EMT, and stemness assays. ***In vivo*:** xenograft models and human CRC samples. **Status:** focused mainly on colorectal cancer	107, 275
**Other prototype ginsenosides**Re and Rf	Tumor growth and cell-cycle regulation	Re: MITF-related melanoma growth inhibition; Rf: G2/M arrest and mitochondrial apoptosis	***In vitro*:** melanoma and osteosarcoma models. ***In vivo*:** zebrafish and xenograft evidence for Re; Rf evidence is mainly cellular. **Status:** model-specific and mechanistically narrow	54, 55
Rg3	Tumor growth and regulated cell death	Cell-cycle arrest; PI3K/Akt and NF-κB inhibition; ROS-associated mitochondrial apoptosis; ER stress and autophagy; context-dependent mTORC1 regulation	***In vitro*:** multiple solid and hematological tumor models. ***In vivo*:** several xenograft and precancerous-lesion models. **Status:** extensive preclinical evidence, but effects vary with stereochemistry, dose, and tumor context	47, 62, 66, 81, 110, 276, 277
	EMT, metastasis, TME remodeling, and angiogenesis	H19- and EMT-related regulation; MMP suppression; inhibition of stemness, VEGF signaling, endothelial activation, and vasculogenic mimicry	***In vitro*:** migration, invasion, stemness, and endothelial assays. ***In vivo*:** xenograft and zebrafish angiogenesis models. **Status:** broad but predominantly preclinical evidence	108, 113, 115, 116, 128, 131, 278
	Antitumor immunity	Immunogenic cell death; EGFR/GSK-3β-mediated PD-L1 degradation; enhanced dendritic-cell, T-cell, and NK-cell activity	***In vitro*:** tumor-immune-cell and checkpoint assays. ***In vivo*:** tumor-bearing animal models. **Status:** among the more developed immune-related ginsenoside mechanisms; clinical immune validation remains limited	134, 135, 135, 140, 203
	Metabolic and epigenetic regulation	H19/miR-324-5p/PKM2, STAT3/HK2, and DNMT3A/miR-532-3p/HK2 axes; regulation of DNA methylation and non-coding RNAs	***In vitro*:** glycolytic and epigenetic studies. ***In vivo*:** selected xenograft validation. **Status:** mechanistic causality and metabolic-flux confirmation vary among studies	156-158, 163
Rh2	Tumor growth and regulated cell death	Akt, PBK/TOPK, Axl, Wnt, NF-κB/MAPK, and PI3K/Akt/mTOR regulation; mitochondrial ROS, ER stress, autophagy, and intrinsic/extrinsic apoptosis	***In vitro*:** extensive evidence across solid and hematological malignancies. ***In vivo*:** multiple xenograft models. **Status:** broad preclinical support; 20S-Rh2 generally shows greater activity than 20R-Rh2	48, 50, 51, 67, 74, 83, 279, 280
	EMT, metastasis, and TME remodeling	EMT and MMP inhibition; IL-6/STAT3 suppression; reduced invasive signaling; M2-to-M1 macrophage repolarization	***In vitro*:** migration, invasion, and macrophage-related assays. ***In vivo*:** selected tumor-bearing models. **Status:** preclinical evidence is supportive but heterogeneous across tumor types	120, 121, 281
	Antitumor immunity	PD-L1 suppression; enhanced NK-cell surveillance through the ERp5/NKG2D/MICA axis	***In vitro*:** checkpoint and NK-cell recognition studies. ***In vivo*:** selected breast and lung tumor models. **Status:** mechanistically defined but supported by relatively few independent studies	139, 141
	Metabolic and epigenetic regulation	HIF-1α/PDK4-mediated shift from glycolysis to oxidative phosphorylation; STAT3/c-Myc inhibition; lncRNA, EZH2, and m⁶A-related regulation	***In vitro*:** metabolic, transcriptomic, and epigenetic studies. ***In vivo*:** limited xenograft validation. **Status:** exposure-effect and PK/PD relationships remain insufficiently defined	68, 71, 155, 167, 168, 282
CK	Tumor growth and regulated cell death	Cell-cycle arrest; PI3K/Akt/NF-κB and Akt/mTOR/c-Myc inhibition; apoptosis, programmed necrosis, FOXO1-related ferroptosis, and STAT3/ER-stress-related death	***In vitro*:** gastric, prostate, liver, and hematological tumor models. ***In vivo*:** selected xenograft and leukemia models. **Status:** strongest support is for regulated cell death	76, 77, 88, 283-286
	Invasion and immune escape	SDF-1/MMP-2/9/PKCα/ERK-related invasion inhibition; STAT3/PD-L1-associated tumor-cell regulation	***In vitro*:** invasion and checkpoint-related assays. ***In vivo*:** limited confirmation. **Status:** substantially less developed than the cell-death evidence	75, 287
	Metabolic and microbiome-associated regulation	Inhibition of glycolysis and glutamine utilization; reduced ATP production; remodeling of gut microbiota	***In vitro*:** glycolytic and glutamine-metabolism studies. ***In vivo*:** AOM/DSS-induced colitis-associated CRC model. **Status:** promising but causal microbiome-efficacy relationships remain unresolved	160, 172, 286
Rg5	Tumor growth and regulated cell death	Akt/Bcl-2 and PI3K/Akt/mTOR inhibition; DNA damage; caspase-dependent apoptosis and autophagy	***In vitro*:** esophageal, retinal, cervical, and breast cancer models. ***In vivo*:** selected xenograft models. **Status:** consistent preclinical activity but limited mechanistic replication	288-292
	EMT and metastasis	NF-κB/EphA2-related migration inhibition; suppression of TGF-β1-induced EMT, MMP-2/9, anoikis resistance, and stem-like properties	***In vitro*:** migration, invasion, and EMT assays. ***In vivo*:** limited animal confirmation. **Status:** anti-metastatic evidence remains less extensive than cell-death evidence	100, 109
Rk1	Tumor growth and regulated cell death	G0/G1 arrest; ER stress; caspase-dependent apoptosis; AMPK/mTOR-mediated cytotoxic autophagy	***In vitro*:** cervical, breast, lung, and liver cancer models. ***In vivo*:** xenograft and chemically induced HCC models. **Status:** multidomain preclinical evidence with limited independent replication	85, 138, 293, 294
	Metastasis, immune escape, and metabolism	Inhibition of EMT and MMP activity; NF-κB/PD-L1 suppression; ERK/c-Myc-mediated inhibition of glutamine metabolism	***In vitro*:** EMT, checkpoint, and metabolic assays. ***In vivo*:** selected tumor models. **Status:** each domain is supported by relatively few studies	109, 138, 159
Rk3	Tumor growth and regulated cell death	PI3K/Akt/mTOR-related cell-cycle arrest, mitochondrial apoptosis, and autophagy	***In vitro*:** lung, liver, and esophageal cancer models. ***In vivo*:** xenograft and chemically induced HCC models. **Status:** evidence is focused mainly on apoptosis and autophagy	86, 87, 295
**Other rare ginsenosides**Rh1, Rg2, and Rg18	Tumor growth, regulated cell death, and metastasis	Rh1: mitochondrial ROS/ER stress and RhoA/ROCK1-related apoptosis; Rg2/Rg18: ROS/AMPK- and cell-cycle-related growth inhibition; Rh1: CK2α-HHEX/CCL20/CCR6-associated metastasis inhibition	***In vitro*:** several tumor-cell models. ***In vivo*:** selected Rh1 tumor models. **Status:** compound-specific evidence with limited mechanistic breadth	296-302
**Other rare ginsenosides**Rh4, F1, and F2	Immune, metabolic, and microbiome-associated regulation	Rh4: HDAC2/JAK/STAT- or Akt-related PD-L1 suppression and microbiota-associated bile acid regulation; F1: IGF-1-dependent NK-cell activation; F2: miR-193a-5p/β-catenin/c-Myc/HK2-mediated glycolysis inhibition	***In vitro*:** checkpoint, NK-cell, and metabolic assays. ***In vivo*:** selected immune and microbiome models. **Status:** evidence is mechanism-specific and remains limited for each compound	136, 137, 142, 162, 171
GPS and related fractions	Direct tumor growth inhibition and regulated cell death	Growth inhibition; mitochondrial apoptosis; NLRP3 activation; autophagy-related radiosensitization	***In vitro*:** breast, bone, and colorectal tumor models. ***In vivo*:** selected tumor-bearing models. **Status:** direct cytotoxic evidence is less extensive than host-mediated evidence	90, 91, 303
	Metastasis and antitumor immunity	Macrophage and NK-cell activation; complement responses; dendritic-cell and cytotoxic T-lymphocyte activation; enhanced anti-PD-1/PD-L1 responses	***In vitro*:** immune-cell activation studies. ***In vivo*:** multiple mouse tumor models. **Status:** broad host-mediated activity, but structural heterogeneity complicates comparison	146-148, 150, 304
	Metabolic and microbiome-associated regulation	Regulation of the kynurenine/tryptophan ratio; remodeling of gut microbial composition	***In vivo*:** tumor-bearing and microbiome-related mouse models. **Status:** causal links among polysaccharide structure, microbiota change, and tumor control remain incompletely defined	150, 173
**Ginsenoside derivatives and related compounds**1C, C3DM, 2-deoxy-Rh2, Rh2E2, Rh2-O, 4-XL-PPD, and related derivatives	Tumor growth and regulated cell death	Cell-cycle inhibition; ROS/ER stress; mitochondrial apoptosis; p53/MDM2 regulation; glycolysis-associated cytotoxicity	***In vitro*:** multiple tumor-cell models. ***In vivo*:** selected xenograft and intracranial tumor models. **Status:** emerging structure-activity evidence; direct potency comparisons remain difficult	161, 305-313
	Metastasis and metabolic regulation	EMT and MMP inhibition; c-Jun/COX2/PGE2-associated invasion suppression; interference with glycolysis and cellular energy metabolism	***In vitro*:** invasion and metabolic assays. ***In vivo*:** limited confirmation for selected derivatives. **Status:** early-stage preclinical evidence	103, 104, 161, 308, 311

**Note:** This table summarizes representative rather than exhaustive evidence for the anticancer activities of major ginseng-derived constituents. Mechanisms were grouped according to the principal biological processes discussed in Sections 3.1-3.5, while individual pathways and targets were retained only when supported by representative experimental studies. Evidence status was qualitatively assessed according to the number of independent studies, diversity of tumor models, availability of in vivo confirmation, genetic or rescue validation, and clinical evidence; it does not represent a formal systematic evidence-grading or risk-of-bias assessment. Statements such as “limited,” “moderate,” or “predominantly preclinical” indicate the current breadth and maturity of evidence rather than the absence of biological activity. References are representative and are not intended to include every published study. CK, Compound K; GPS, ginseng polysaccharides; PPD, protopanaxadiol; PPT, protopanaxatriol; TME, tumor microenvironment; EMT, epithelial-mesenchymal transition; MMP, matrix metalloproteinase; ROS, reactive oxygen species; ER, endoplasmic reticulum; NK, natural killer; CRC, colorectal cancer; HCC, hepatocellular carcinoma; PK/PD, pharmacokinetics/pharmacodynamics; AOM/DSS, azoxymethane/dextran sulfate sodium; m6A, N6-methyladenosine; lncRNA, long non-coding RNA.

**Table 3:** Representative delivery systems, pharmacokinetic limitations, and translational relevance of ginseng-derived agents

Agent	Main barrier	Delivery strategy	Key advantage	Translational relevance	Ref.
Rg3	Poor solubility, low permeability, and limited bioavailability	Rg3 proliposomes, Rg3-based liposomes	Improves dispersion, formulation state, oral bioavailability, and tumor/TME delivery	Supports Rg3-related apoptosis, anti-angiogenesis, immune regulation, and chemosensitization	240, 242
	Limited tumor accumulation and insufficient co-delivery efficiency	PEG-GO-FA/ICG-Rg3, Rg3-PLGA@TMV, Rg3-PTX-LPs, G/R-MLP	Enhances tumor targeting, controlled release, photodynamic effect, and co-delivery	Strengthens cancer stemness inhibition, TME remodeling, anti-metastatic activity, and combination chemotherapy	215, 216, 241, 243
Rh2	Poor solubility, intestinal metabolism, P-gp-related efflux, and limited tumor exposure	Rh2-functionalized liposomes, PTX-Rh2-lipo, LTL@Rh2@Lipo-GE11, Rh2-containing cocktail liposomes	Provides nanoscale delivery and combines delivery enhancement with the intrinsic antitumor activity of Rh2	Enhances apoptosis induction, antimetastatic activity, immune modulation, and chemotherapy combination	214, 244, 245, 247
	Poor aqueous solubility and limited cellular uptake	Rh2 nanoniosomal formulation, BSA-Rh2 NPs, Rh2-M	Improves solubility, stability, cellular uptake, and tumor targeting	Enhances antitumor efficacy in prostate, lung, colon, and other cancer models	219, 220, 251
CK	Poor aqueous solubility, insufficient absorption, and limited tissue retention	CK mixed micelles, APD-CK micelles	Improves solubilization, drug loading, retention, cellular uptake, and liver targeting	Supports CK-mediated apoptosis and liver cancer-targeted delivery	211, 250
	Poor solubility and limited delivery efficiency	CK-OCMC NPs, DCY51T-AuCK NPs, GCKT-Lipo	Enhances water solubility, cellular uptake, formulation stability, and photothermal synergy	Strengthens caspase-mediated apoptosis and combination antitumor efficacy	225, 226, 248
Rh1 / Rh2	Low oral bioavailability related to intestinal metabolism and efflux transport	Rh1-SMEs, ECG-MEs	Improves oral absorption, systemic exposure, and multicomponent delivery	Supports systemic antitumor exposure and enhances Rh2-related apoptosis in xenograft models	253, 254
Rg3 / GLP / oridonin	Need for multicomponent oral delivery and TME-related modulation	RGO-SMEDDS	Improves self-emulsifying delivery and multicomponent exposure	Supports immune restoration, anti-angiogenesis, and EGFR/Akt/GSK-3-related HCC inhibition	255
Rg5	Poor solubility and weak tumor selectivity	FA-Rg5-BSA NPs	Improves water dispersion, folate receptor-mediated uptake, and tumor targeting	Supports Rg5-related antiproliferative and pro-apoptotic activity	221
Rh1 / Rh2/PPD	Poor aqueous solubility and limited circulation stability	PEG-Rh1/Rh2 conjugates, PEG-PPD conjugates	Improves aqueous dispersion, circulation time, pH-dependent release, and tumor-site delivery	Enhances the anticancer potential of aglycone- or rare ginsenoside-based agents	217, 218
Rg1 / Rb1 / PPD combination	Limited tumor selectivity, insufficient co-delivery, and chemotherapy-related toxicity	Dox@Rg1 NPs, Rb1/PTX/PPD co-loaded GPP NPs	Improves tumor targeting, co-delivery efficiency, and reduces off-target toxicity	Enhances chemotherapy efficacy and supports TME modulation	222, 223
GsE / ginseng extract	Limited tumor retention and insufficient TME-directed delivery	ICG/CMSN@GsE, GDNPs	Enhances tumor retention, macrophage specificity, and immune/TME regulation	Links delivery improvement with macrophage reprogramming, T-cell activation, and cold-to-hot TME conversion	93, 224, 236-238
Ginseng-derived exosome-like nanoparticles	Limited tissue penetration and glioma targeting	GENs	Crosses the BBB and improves glioma-targeted delivery	Inhibits glioma progression and regulates tumor-associated macrophages	239
Ginsenoside-based CDs, Re-based CDs, Ginseng-extract-derived CDs	Limited cellular uptake and need for low-toxicity nanoscale delivery	GS-CDs, Re-CDs, GCDs	Improves cellular uptake, provides low toxicity, and supports bioimaging	Promotes apoptosis and ferroptosis; Re-CDs improve Re uptake	231-233
Rb3 / Rb1 / Rg1	Limited intracellular delivery and insufficient immunotherapy-related activity	Rb3-CNT, Rb1/Rg1-CNT	Enhances drug loading, cellular interaction, and antiproliferative activity	Supports TNBC immunotherapy-related activity and improves anticancer effects	234, 235
Ginseng extracts / CK / plant-mediated preparations	Limited stability, weak delivery efficiency, and need for multifunctional platforms	GBAu NPs, GBAg NPs, AgNPs, P.g AgNPs, HGRCm-ZnO NPs, DCY51T-AuCK NPs	Provides nanoscale delivery and adds photothermal, antioxidant, or apoptotic effect	Supports extract-based antitumor activity, apoptosis induction, and potential combination therapy	226-230

**Note:** This table presents representative rather than exhaustive delivery systems and links the principal physicochemical or pharmacokinetic limitations of each ginseng-derived agent with the corresponding formulation strategy, reported pharmaceutical advantages, and potential translational relevance. Most evidence is derived from preclinical studies; therefore, the translational relevance described here indicates experimentally supported potential rather than established clinical benefit. Formulation abbreviations are retained from the original studies. CK, Compound K; TME, tumor microenvironment; P-gp, P-glycoprotein; NP, nanoparticle; PEG, polyethylene glycol; GO, graphene oxide; FA, folic acid; ICG, indocyanine green; PLGA, poly(lactic-co-glycolic acid); TMV, tumor-derived microvesicle; PTX, paclitaxel; BSA, bovine serum albumin; OCMC, O-carboxymethyl chitosan; TPGS, D-α-tocopheryl polyethylene glycol 1000 succinate; SME, self-microemulsion; ME, microemulsion; SMEDDS, self-microemulsifying drug delivery system; GLP, Ganoderma lucidum polysaccharide; GDNP, ginseng-derived nanoparticle; GEN, ginseng-derived exosome-like nanoparticle; BBB, blood-brain barrier; HCC, hepatocellular carcinoma.

## Abbreviations

ABC: ATP-binding cassette
AMPK: AMP-activated protein kinase
Bcl-2: B-cell lymphoma 2
BCRP: breast cancer resistance protein
CDs: carbon dots
CK: compound K
CNTs: carbon nanotubes
CRC: colorectal cancer
DDS: drug delivery systems
EGFR: epidermal growth factor receptor
EMT: epithelial-mesenchymal transition
GBM: glioblastoma
GDNPs: ginseng-derived nanoparticles
GENs: ginseng-derived exosome-like nanoparticles
GLP: *Ganoderma lucidum* polysaccharide
GLUT1: glucose transporter type 1
GPS: ginseng polysaccharides
HCC: hepatocellular carcinoma
HIF-1α: hypoxia-inducible factor 1-alpha
HK2: hexokinase 2
LAMC2: laminin subunit gamma 2
LDHA: lactate dehydrogenase A
m6A: N6-methyladenosine
MAPK: mitogen-activated protein kinase
MDR: multidrug resistance
MM: myeloma
MMPs: matrix metalloproteinases
MRP1: multidrug resistance-associated protein 1
mTOR: mechanistic target of rapamycin
NF-κB: nuclear factor-κB
NK: natural killer cells
NPs: nanoparticles
NSCLC: non-small-cell lung cancer
OA: oleanolic acid
P-gp: P-glycoprotein
PCa: prostate cancer
PD-1: programmed cell death protein 1
PD-L1: programmed death-ligand 1
PFKFB3: 6-phosphofructo-2-kinase/fructose-2,6-bisphosphatase 3
PI3K: phosphatidylinositol 3-kinase
PKM2: pyruvate kinase isozyme type M2
PPD: protopanaxadiol
PPT: protopanaxatriol
ROS: reactive oxygen species
STAT3: signal transducer and activator of transcription 3
TCM: traditional Chinese medicine
TGF-β: transforming growth factor-β
TME: tumor microenvironment
VDAC1: voltage-dependent anion channel 1
VEGF: vascular endothelial growth factor
